# Nanoencapsulation of Pomegranate Extract to Increase Stability and Potential Dermatological Protection

**DOI:** 10.3390/pharmaceutics13020271

**Published:** 2021-02-17

**Authors:** Lucía Yepes-Molina, José A. Hernández, Micaela Carvajal

**Affiliations:** 1 Aquaporins Group, Centro de Edafología y Biología Aplicada del Segura (CEBAS-CSIC), Campus de Espinardo, E-30100 Murcia, Spain; lyepes@cebas.csic.es; 2 Biotechnology of Fruit Trees Group, Centro de Edafología y Biología Aplicada del Segura (CEBAS-CSIC), Campus de Espinardo, E-30100 Murcia, Spain; jahernan@cebas.csic.es

**Keywords:** pomegranate, antioxidant capacity, membrane vesicles, cauliflower, keratinocytes, oxidative stress, cytotoxicity

## Abstract

Pomegranate extract (PG-E) has been reported to exert a protective effect on the skin due to its antioxidant activity. Ingredients rich in phenolic compounds are unstable in extract solutions, and, therefore, the use of a suitable nanosystem to encapsulate this type of extract could be necessary in different biotechnological applications. Thus, we investigated the capacity of *Brassica oleracea* L. (cauliflower) inflorescence vesicles (CI-vesicles) to encapsulate PG-E and determined the stability and the antioxidant capacity of the system over time. In addition, the protective effect against UV radiation and heavy metals in HaCaT cells was also tested. The CI-vesicles had an entrapment efficiency of around 50%, and accelerated stability tests did not show significant changes in the parameters tested. The results for the HaCaT cells showed the non-cytotoxicity of the CI-vesicles containing PG-E and their protection against heavy metals (lead acetate and mercuric chloride) and UV-B radiation through a reduction of oxidative stress. The reduction of the percentage of deleted mtDNA (mtDNA4977, “common deletion”) in UV-treated HaCaT cells due to the presence of CI-vesicles containing PG-E indicated the mechanism of protection. Therefore, the effects of CI-vesicles loaded with PG-E against oxidative stress support their utilization as natural cosmeceuticals to protect skin health against external damage from environmental pollution and UV radiation.

## 1. Introduction

Pomegranate (*Punica granatum* L.), a fruit of the *Punicaceae* family, is considered a fruit with high pharmaceutical value since its bioactive compounds have been shown to have biological activities in the treatment of several human diseases [1]. The main benefit is due to the antioxidant potential derived from the high concentrations of phenolic compounds, such as galloylglucose, punicalagin, punicalin, ellagic acid, and gallic acid [2,3]. Besides, anthocyanins and other nutraceutical components, such as sterols, γ-tocopherol, punicic acid, and hydroxybenzoic acids, have been found in the different parts of pomegranate [4,5]. Thus, functional products enriched with pomegranate extract (PG-E) have been reported to be useful for the treatment of certain diseases—such as diabetes mellitus, obesity, and cardiovascular and gastrointestinal diseases [1,6,7]—since their antioxidant potential gives protection from inflammation because it reduces the activity of cytokines, such as tumor necrosis factor-α (TNF-α) or interleukin-6 (IL-6) [8,9,10], as well as the levels of total cholesterol, low density lipoprotein (LDL), and lipid peroxidation [11]. Further, beneficial and protective effects of PG-E in the skin are also due to antioxidant activity, as reported in different studies [12,13]. In this regard, it is important to focus on the keratinocytes, as they comprise much of the outermost layer of skin (epidermis) [14]. Therefore, keratinocytes suffer damage due to extrinsic stimuli (UV exposure or pollutants, such as heavy metals) [15,16]. These stimuli trigger an excessive production of reactive oxygen species (ROS), which entails a loss of cellular functions and even cell death [17,18]. It is well known that ROS is a threat to cellular integrity, as it causes damage to essential macromolecules, including DNA, lipids, and proteins [19]. Regarding DNA damage, it has been observed to be more persistent in mitochondrial DNA (mtDNA) than in nuclear DNA due, among other causes, to limited repair mechanisms [20]. An indicator of DNA damage is a large deletion of 4977 bp from mtDNA called “common deletion”, which is considered an early marker for mutations induced by high levels of ROS [21,22]. It has been reported that PG-E can reduce the H_2_O_2_ overproduction as well as the cytotoxicity and the inflammatory stress induced by UV exposure [13,23].

Based on the above, this type of extract is of great interest as a natural cosmeceutical for skin health. But, one problem is that phenolic compounds are unstable in extract solutions, and, therefore, it is necessary to remove the solvents of the extracts to stabilize them. The shelf life of the phenolics could be enhanced in the dry extracts, but the stability of the formulated liquid extracts is very limited. Thus, procedures to prolong the stability of the final product, such as the addition of pectins for jelly formation, have been investigated [24]. Similarly, microencapsulation has been reported as a suitable option to stabilize the phenolics of the PG-E [25]. In this procedure, the phenolics are surrounded by a maltodextrin matrix in order to produce small capsules, with significant improvement of the antioxidant and α-glucosidase inhibitory activities.

Recently, new technologies of encapsulation, such as the use of membrane vesicles derived from natural sources, have been studied for different applications, such as cosmetics or therapy, such as treatment of colitis or melanoma [26,27,28,29,30]. The most profitable sources may well be those of plant origin since, in many crops, by-products are produced, which can be used to obtain membrane vesicles. The latest research in our group has focused on the study of stable natural membrane vesicles from brassicas. Plasma membrane vesicles from broccoli (*Brassica oleracea* L. var. italica) are characterized by their potential to stabilize the bioactive glucosinolate glucoraphanin [31]; the stability of this type of vesicle was studied in other work [32] and was found to be related to aquaporins. Recent work confirmed the potential of these vesicles as carriers in cosmetic or therapeutic applications [28]. Besides, in this study, an interaction between plant and human cell membranes was shown, revealing their potential in numerous applications in nanotechnology. In addition to broccoli-derived vesicles, membrane vesicles from cauliflower inflorescence have been well characterized [33]. The vesicles described in this study had sizes between 300 and 400 nm, appropriate for use in various biotechnological applications [34]. Besides, the osmotic permeability (*Pf*) values are related to vesicle functionality and membrane integrity, and high values of *Pf* have been determined in vesicles from *Brassica oleracea* L. var. botrytis inflorescences [33]. These types of vesicles are defined by their versatility since, in addition to their use in cosmetics, applications in agriculture are being studied [35,36]. All these findings lead us to propose these membrane vesicles as nanocarriers, whose advantages are based on their specific lipid/protein composition, their biodegradability, and their ability to carry the encapsulated substance to the target cells.

Therefore, the objective of this work was to test the capacity of *B. oleracea* var. botrytis inflorescence vesicles (cauliflower inflorescence vesicles: CI-vesicles) to encapsulate PG-E and to determine the stability of the vesicles-extract system and its antioxidant capacity over time. Besides, the protective effect of PG-E against UV radiation and heavy metals was determined in a cell line of immortalized keratinocytes (HaCaT), and the protective mechanism was studied through mtDNA^4977^ deletion.

## 2. Materials and Methods

### 2.1. Materials

*Brassica oleracea* L. var. botrytis inflorescences were collected from a commercial farm sited in the Region of Murcia (Lorca, Murcia, Spain), and pomegranate extract (PG-E) was obtained from MitraSol Technologies S.L. (NUTRAGRANATE^®^, Elche, Alicante, Spain).

### 2.2. Cauliflower Inflorescences Vesicles (CI-Vesicles)

Cauliflower inflorescences were cut into small pieces before vacuum-filtering, at a 1:1.6 (*w/v*) ratio, with an extraction buffer (0.5 M sucrose, 1 mM DTT, 50 mM HEPES, and 1.37 mM ascorbic acid, at pH 7.5) and 0.5 g of Polyvinylpyrrolidone (PVP). The mixture was homogenized using a blender and filtered through a nylon mesh (pore diameter of 100 µm). The filtrate was centrifuged at 10,000× *g* for 30 min at 4 °C. The supernatant was recovered and centrifuged for 35 min at 100,000× *g* and 4 °C, and the pellet obtained was suspended in 500 µl of FAB buffer (5 mM PBS and 0.25 M sucrose, pH 6.5) for storage at −80 °C. The protein concentration in this microsomal fraction was determined by the Bradford method [37], using bovine serum albumin as the standard.

### 2.3. Particle Size, Zeta Potential, and Polydispersity Index Analysis of Cauliflower Inflorescences Vesicles (CI-Vesicles)

Dynamic light scattering (DLS) was used to detect particle size, zeta potential, and polydispersity index at a temperature of 20 °C using a Zetasizer Nano (Malvern Instruments, Malvern, UK) in a similar way as previously was reported [38,39,40]. Transmission electron microscopy (TEM) was performed as described previously [41].

### 2.4. Pomegranate Extract Encapsulation and Entrapment Efficiency (EE)

**Figure 1:**
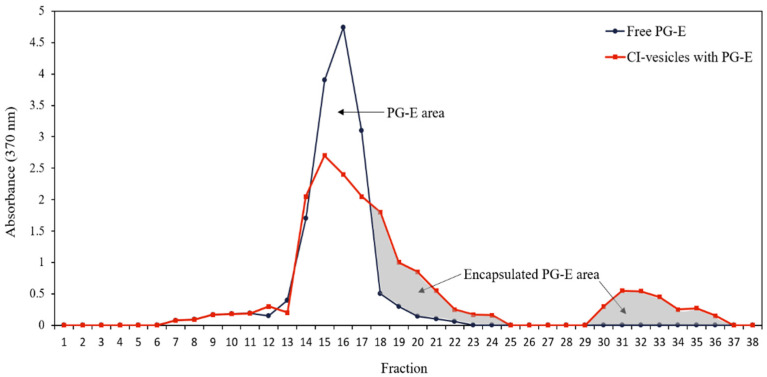
Absorbance (370 nm) of each fraction obtained after passing through a Sephadex column the samples of free PG-E (blue line) and CI-vesicles with encapsulated PG-E (red line). The grey area indicates the proportion of PG-E encapsulated in CI-vesicles. PG-E, pomegranate extract; CI-vesicles, cauliflower inflorescence vesicles.

For encapsulation of the PG-E, enriched in punicalagin, in CI-vesicles, the microsomal fraction pellet was resuspended in FAB buffer containing 30% PG-E. The mixture was shaken vigorously and then stabilized in a glycerol solution, with a concentration of 0.015% (*w*/*w*) protein and 0.15% (*w*/*w*) PG-E. Free PG-E was prepared in the same way but without CI-vesicles. For the experiments conducted, different concentrations were produced based on the initial concentration. The entrapment efficiency (EE) was measured with a method based on the separation of particles by size and physicochemical nature in Sephadex G25 columns. The columns were equilibrated at room temperature with a solution of glycerol:water (1:3) at least 3 h before use. PG-E, free in glycerol and encapsulated in CI-vesicles in glycerol (2.5 mL), was loaded onto the Sephadex columns after dilution in water (1:3). The samples were eluted by passing 10 mL of glycerol:water (1:3) solution and 5 mL of 0.2 N NaOH to disrupt the vesicles. Fractions of 1 mL were collected to measure the protein content and the absorbance at 370 nm to determine the PG-E content. The absorbance (370 nm) corresponding to each fraction (1–38) is represented in Figure 1, and the area under the graph was calculated with the program Image J [42]. EE percentage was calculated through a relationship between the total area (considered 100%) and the encapsulated area. The experiments were performed in triplicate.

### 2.5. Protein Content

The protein content in CI-vesicles with PG-E was measured by the Bradford method [37]. Measurements were carried out every 15 days on samples stored at 20 °C, 4 °C, or 40 °C for 3 months.

### 2.6. Color

The color of the product (CI-vesicles with PG-E) was determined by measuring the visible absorbance spectrum. The maximum absorbance of the samples in the visible spectrum was at 370 nm. Measurements were carried out every 15 days on samples stored at 20 °C, 4 °C, or 40 °C for 3 months.

### 2.7. Antioxidant Capacity

The antioxidant capacity was determined by the DPPH radical (2,2-diphenyl-1-picryldhydrazyl) assay [43,44]. The DPPH solution was prepared in methanol and had an absorbance of 1 at 517 nm. A 96-well plate was used to carry out the assay. An initial measurement at 517 nm was made for 250 µL of DPPH solution, using methanol as the blank. Two microliters of each sample or standard (prepared with Trolox) were added to the wells containing the DPPH solution. The plate was shaken, left for 30 min in the dark, and the final absorbance was measured again at 517 nm. To determine the stability of the antioxidant activity of the CI-vesicles with PG-E, measurements were carried out every 15 days on samples stored at 20 °C, 4 °C, or 40 °C for 3 months.

### 2.8. HaCaT Cells Culture

HaCaT cells, a spontaneously immortalized human keratinocyte line [45], were cultured in Dulbecco’s Modified Eagle’s Medium (DMEM) with 10% FBS, 1% penicillin-streptomycin, and 1% l-glutamine at 37 °C and 5% CO_2_. The subcultures were carried out when the cells reached 70–90% confluence.

### 2.9. Applied Treatments and Stresses (Heavy Metals and UV-B Radiation)

When the cells reached 60–70% confluence, they were washed with PBS buffer (37 °C), and treatments with PG-E, in CI-vesicles and free (0.01, 0.005, 0.001, and 0.0005%, prepared using 0.15% *w/w* PG-E in glycerol), were applied for 24 h. Then, the cells were subjected to the stresses. Different concentrations of lead acetate and mercuric chloride (0 µM, 90 + 30 µM, 500 + 50 µM, 1000 + 70 µM, and 2000 + 90 µM) were applied for 24 h. HaCaT cells were exposed to UV radiation (20 J/cm^2^) using a Bio-Link Crosslinker BLX 312. Control cells were incubated in parallel without irradiation.

### 2.10. Cell Viability (MTT Assay)

The effect of different concentrations of PG-E free and encapsulated in CI-vesicles and of stresses on the viability of HaCaT cells was determined by the MTT (3-(4,5-dimethylthiazol-2-yl)-2,5-diphenyltetrazolium bromide) assay [46]. Cells were plated at 3200 cells/well in 198 µL of DMEM complete medium in a 96-well plate and were cultivated at 37 °C and 5% CO_2_ until 60–70% confluence. Then, 2 µL of different dilutions of samples was added to the wells (each sample was repeated in 6 wells), and, after 24 h of incubation, cell viability was determined. Briefly, 200 µL of MTT (1 mg/mL in DMEM) was added after complete removal of the medium from the wells, and the cells were incubated for 4 h at 37 °C and 5% CO_2_. The MTT solution was then removed and replaced with 100 µL of DMSO, and the plate was shaken. The absorbance at 570 nm was recorded using a microplate reader (BMG Labtechnologies, Fluostar Omega, Ortenberg, Germany). The percentage viability of the treated cells and the protection from mortality were calculated as follows:

(1) Cell viability (%)= (Abs570nm)sample (Abs570nm)control × 100

(2) $$\mathrm{Protection}\;\mathrm{from}\;\mathrm{mortality}\;\left(\%\right)=100-{\left(\frac{{\;\mathrm{Cell}\;\mathrm{mortality}}_{\mathrm{treated}}\;\left(\%\right)\;\times 100\;}{{\mathrm{Cell}\;\mathrm{mortality}}_{\mathrm{untreated}}\;\left(\%\right)}\right)}_{\;}$$

(3) Cell mortality (%)=100−cell viability (%)

### 2.11. Lipid Peroxidation Levels

Lipid peroxidation was evaluated, with the thiobarbituric acid reactive substances (TBARS) assay [47], in the cell culture medium and cells after UV radiation exposure. The treated cells were washed with cooled PBS, scraped into trichloroacetic acid (TCA) (2.8%, *w*/*v*), and disrupted by grinding with a pestle. Total protein was determined by the Bradford assay [37]. The cell suspension or cell culture medium was mixed with thiobarbituric acid (TBA) (0.5% *w*/*v*) in TCA (20% *w*/*v*), heated (20 min, 90 °C), and centrifuged (10,000 rpm, 5 min). TBA reacts with the oxidative degradation products of lipids to yield red complexes that absorb at 532 nm. The amount of TBA reactive substances was determined using a spectrophotometer, and the absorbance at 600 nm was subtracted to eliminate the effect of turbidity. The amount of the malondialdehyde (MDA)-TBA complex present was calculated using an extinction coefficient (ε) of 155 mM^−^^1^ cm^−^^1^.

### 2.12. DNA Extraction and Analysis and Quantitative PCR

Genomic DNA extraction from HaCaT cells was performed using the QIAamp^®^ DNA Micro Kit (Qiagen, Hilden, Germany) according to the manufacturer’s protocols. These ensured the isolation of total (nuclear and mitochondrial) DNA. The total DNA concentration was determined using a NanoDrop 2000 spectrophotometer (Thermo Fisher Scientific, Wilmington, DE, USA).

Quantitative PCR was carried out using an Applied Biosystems™ 7500 Real-Time PCR System (Thermo Fisher Scientific), in 10-μL assay volumes, with 2X Power SYBR Green PCR Master Mix (Applied Biosystems, Carlsbad, CA, USA) and a ROX passive reference dye. The volumes and concentrations for the SYBR Green reaction mixes were 5 μL of SYBR Green reaction mix, 0.5 μM forward and reverse primer, and 10 ng of DNA template. The primer sequences are listed in Table 1. The amplification conditions were 2 min at 50 °C and 10 min at 95 °C, followed by 40 cycles of 15 s at 95 °C and 1 min at 60 °C. Quantitative PCR reactions were carried out using primers designed to amplify a highly conserved region of mtDNA essential for cell survival and another region known to be affected by large-scale deletions due to sunlight exposure, the mtDNA^4977^ deletion, in a similar way to Koch et al. [48]. Reactions were also carried out to quantify the nuclear housekeeping gene Beta-actin, which was chosen because it has been validated for human skin DNA analysis and has been found to be stable under these conditions [49].

By comparing the cycle threshold (Ct) number of the mtDNA^4977^ with the average Ct number of the nuclear housekeeper (Beta-actin), the average number of deletion-bearing mtDNA genomes could be calculated in a manner similar to that shown in Powers et al. [50]: A) Efficiency of Beta-actin ^ Ct of Beta-actin for sample/Efficiency of mtDNA^4977^ ^ Ct of mtDNA^4977^ for sample; B) Efficiency of Beta-actin ^ Ct of Beta-actin for sample/Efficiency of mitochondrial conserved region ^ Ct of the conserved region for sample; C) Percentage of genomes carrying the mtDNA^4977^ = ((A × 100)/B).

### 2.13. Statistical Analyses

R software [51] was used to analyze all the data. When multiple comparisons were performed, evaluation involved one-way or two-way ANOVA, followed by the Tukey HSD test or Student t-test. Differences were considered to be significant at *p <* 0.05. All results are presented as the means ± SE.

## 3. Results

### 3.1. Physicochemical and Morphological Characterization

Table 2 shows DLS analysis performed in a similar way to that previously reported [38,39,40]. The CI-vesicles had an average hydrodynamic diameter around 620.7 nm, which increased when PG-E was encapsulated in CI-vesicles (797.5 nm). TEM picture of the shape of CI-vesicles with PG-E is shown in Supplemental material (Figure S1). Zeta potential values of −21 mV were obtained in both CI-vesicles and CI-vesicles with PG-E, indicating adequate stability of the formulations [52] and a negative electric charge on the surface of the vesicles. Regarding free PG-E, it was not possible to measure the size by DLS, and the zeta potential value was −15 mV.

### 3.2. Pomegranate Extract Entrapment Efficiency (EE)

The entrapment efficiency (EE) was determined by absorbance measurements at 370 nm, the wavelength in the visible spectrum at which the absorbance by samples containing PG-E is maximum. Both free PG-E and PG-E encapsulated in CI-vesicles were passed through a Sephadex column, and different fractions were collected to measure the absorbance. The CI-vesicles were disrupted to allow the release of the encapsulated extract. Figure 1 shows the absorbance at 370 nm of different fractions collected after passing free PG-E and CI-vesicles containing PG-E through a Sephadex column. The free PG-E appeared in fractions 13 to 19. For the samples with vesicles, absorbance also appeared from fraction 13 but remained until fraction 24, with a second peak between fractions 30 and 36. The colored areas correspond to fractions where proteins appeared, that is, those fractions containing the CI-vesicles with encapsulated PG-E. The first colored area corresponds to small vesicles that appeared together with the last molecules of the free PG-E, and the second area corresponds to vesicles retained in the column and disrupted by chloroform.

The data regarding the areas under the curves and the protein concentration are shown in Table 3. Taking into account the total area under the free PG-E curve and corresponding to fractions with proteins, an EE of 46.50 ± 1.62% was estimated. No significant differences appeared between the total areas under the curve of the two samples, and, therefore, no extract residues were retained in CI-vesicles without being determined. Besides, the sum of the protein contents of all the fractions collected was the same as the protein content in the sample previous to elution through the Sephadex column. Thus, both the entire extract and all the vesicles passed through the column.

### 3.3. Stability of CI-Vesicles with Encapsulated PG-E

**Figure 2:**
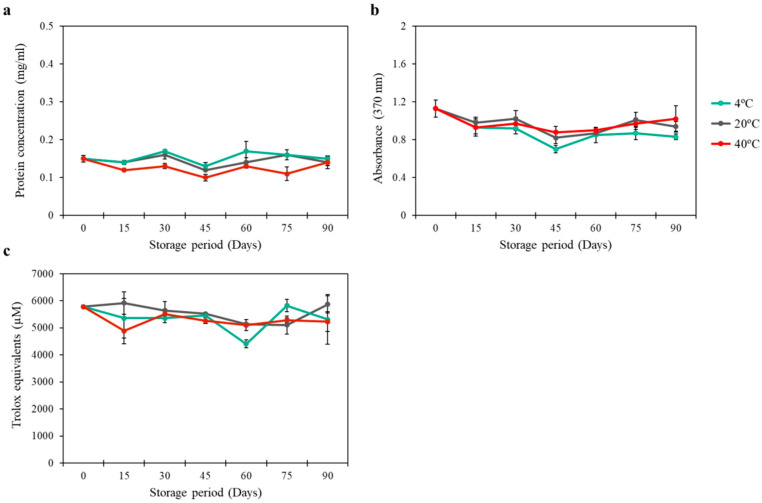
Protein (mg/mL) stability (**a**), color (absorbance at 370 nm) stability (**b**), and antioxidant activity (µM Trolox Equivalents (TE)) (**c**). Measurements were carried out over 90 days, at 4 °C (blue), 20 °C (grey), and 40 °C (red), for cauliflower inflorescence vesicles (CI-vesicles) with encapsulated PG-E. Data are means ± SE (*n =* 3).

The stability of the CI-vesicles with encapsulated PG-E was measured in different ways: (1) protein concentration, (2) color of the solution, and (3) antioxidant activity. The concentration of proteins in the vesicles was measured over 3 months under different storage conditions, and no significant changes were observed in the amount of protein in the product over time under any of the storage conditions (Figure 2a). Besides, the color of the product (CI-vesicles with encapsulated PG-E) was followed for the same storage time and temperature conditions, and no significant changes in the absorbance at 370 nm (the wavelength of maximum absorbance in the visible spectrum for the product) were observed in any sample (Figure 2b). The antioxidant activity was measured every 15 days for 3 months, and no significant differences were recorded (Figure 2c). The stability of the free extract was also determined with no differences in absorbance and trolox equivalent (TE) for over 90 days at different temperatures. Besides, similar values to those obtained from the encapsulated samples were observed (data not shown). The release with chloroform was performed with similar results as with ethanol used in the measurement of antioxidant activity (Table S1).

### 3.4. Antioxidant Activity

The antioxidant activity of PG-E diluted in glycerol (free) and encapsulated in CI-vesicles was determined, and no significant differences were found. Thus, encapsulation did not affect the antioxidant properties of PG-E. Besides, the antioxidant activity of CI-vesicles without PG-E was determined, and no activity was measured (Table 4).

**Figure 3:**
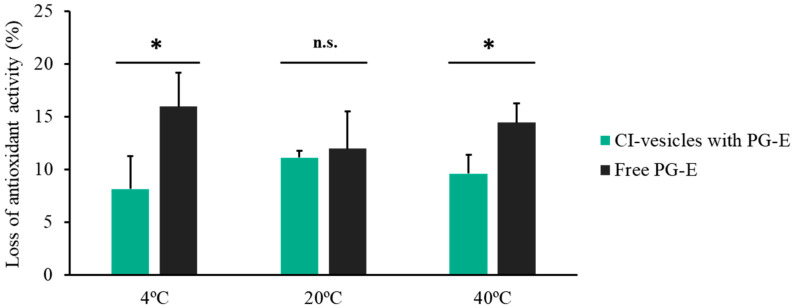
Loss of antioxidant activity (%) with respect to the initial time for pomegranate extract (PG-E), encapsulated in CI-vesicles or free, after 90 days of storage at 4 °C, 20 °C, or 40 °C. Data are means ± SE (*n =* 3). *, significant differences compared to the respective control (*p <* 0.05). n.s., not significant.

Figure 3 shows the percentage loss of antioxidant activity of PG-E, both free and encapsulated in CI-vesicles, after 90 days of storage at different temperatures. At 4 °C and 40 °C, surprisingly similar results were obtained, with a loss of 8.13% and 9.60%, respectively, of the antioxidant activity of PG-E encapsulated in CI-vesicles. Regarding free PG-E, the losses were also similar at 4 °C and 40 °C: 15.96% and 14.45%, respectively. At room temperature (20 °C), no significant differences were found between the loss of antioxidant capacity of the free and encapsulated extract; in both cases, around 11% was lost. Hence, the storage temperature did not influence the loss of antioxidant capacity of the free or encapsulated extract.

### 3.5. Cytotoxic Effects in HaCaT Cells of PG-E Encapsulated in CI-Vesicles

**Figure 4:**
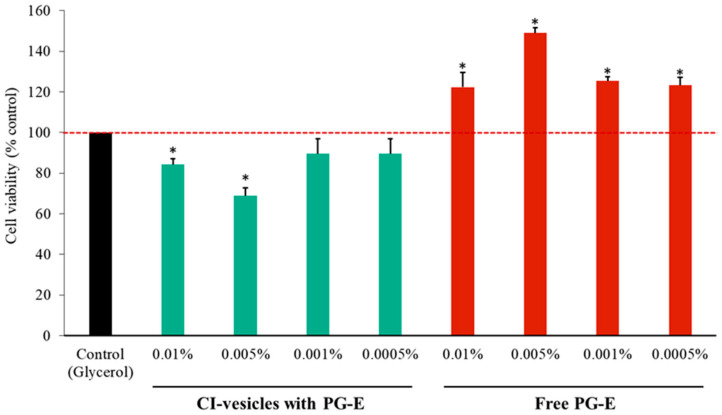
Viability of HaCaT cells incubated for 24 h with different concentrations of pomegranate extract (PG-E), encapsulated in cauliflower inflorescence vesicles (CI-vesicles) or free, as determined by the MTT assay. The results were normalized as a function of the glycerol cytotoxicity. Data are means ± SE (*n =* 6). * *p <* 0.05, significantly different compared to the control group.

The cytotoxic effects of CI-vesicles with encapsulated PG-E were evaluated at different concentrations (0.01%, 0.005%, 0.001%, and 0.0005%), in comparison with free PG-E, 24 h after application, in HaCaT cells. As shown in Figure 4, CI-vesicles with PG-E at the two highest concentrations showed significant differences with respect to the control, but cell viabilities around 80% are not considered cytotoxic [53]. On the other hand, the application of free PG-E produced a significant increase in the viability of HaCaT cells, to values higher than the control. Hence, neither the free nor the encapsulated extract, at the applied concentrations, decreased the viability of HaCaT cells.

**Figure 5:**
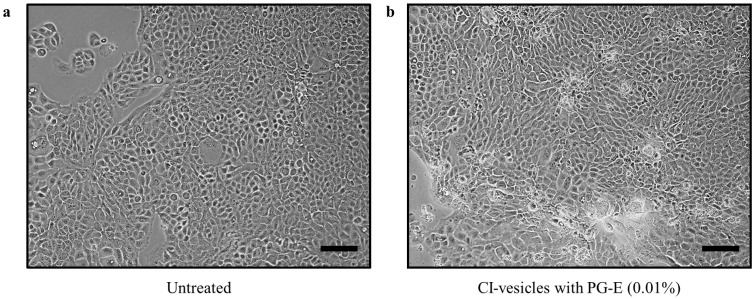
Phase-contrast microscopy images of HaCaT cells: untreated (**a**) and treated for 24 h after reaching 60–70% confluence with cauliflower inflorescence vesicles (CI-vesicles) containing pomegranate extract (PG-E) at a concentration of 0.01% (**b**). Scale bars = 100 µm.

Furthermore, at the morphological level, no changes were seen after the treatment of HaCaT cells with CI vesicles containing PG-E; the cells were tightly connected, exhibited regular morphology, and had the same abundance compared to control cells (Figure 5).

### 3.6. Effect of CI-Vesicles Containing PG-E Against Heavy Metals in HaCaT Cells

**Figure 6:**
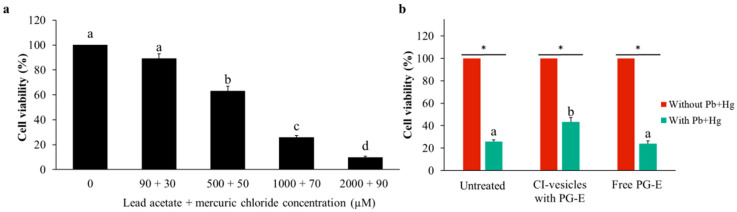
Viability (determined by the MTT assay) of HaCaT cells incubated for 24 h with different combinations of lead acetate and mercuric chloride (µM) (**a**) and treated for 24 h with cauliflower inflorescence vesicles (CI-vesicles) with pomegranate extract (PG-E) or free PG-E previous to the application of heavy metals (1000 µM lead acetate + 70 µM mercuric chloride) (**b**). The results were normalized as a function of the control cytotoxicity. Data are means ± SE (*n =* 6). Different letters indicate significant differences between groups. *, significant differences compared to the respective control (*p <* 0.05).

After testing different concentrations of lead acetate and mercuric chloride (data not shown), distinct combinations of both compounds at different concentrations were chosen for application to HaCaT cells in order to determine the protective effect of CI-vesicles with PG-E against heavy metals. The effect of these combinations of heavy metals on the viability of HaCaT cells was dose-dependent, as shown in Figure 6a. The lowest concentration (90 µM lead acetate +30 µM mercuric chloride) did not affect cell viability, whereas the highest concentration (2000 µM lead acetate + 90 µM mercuric chloride) showed strong cytotoxicity. The effect of PG-E on the cell mortality caused by a combination of lead acetate and mercuric chloride was tested in HaCaT cells by applying 1000 µM lead acetate +70 µM mercuric chloride for 24 h after treatment with PG-E, free or encapsulated in CI-vesicles, also for 24 h. The chosen heavy metal concentrations caused high cytotoxicity but allowed for a possible improvement (Figure 6a). Figure 6b shows the viability of HaCaT cells after treatment with PG-E (in CI-vesicles or free) and then the heavy metals. A significant increase in viability appeared when CI-vesicles containing PG-E were applied, compared to the viability of untreated cells and cells treated with free PG-E.

A summary of the effects of the CI-vesicles with PG-E on the viability of HaCaT cells under heavy metals stress is shown in Table 5. A 17% increase in cell viability occurred with the CI-vesicles containing PG-E, but no increase was detected for cells treated with free PG-E. Treatment of cells with CI-vesicles containing PG-E gave 27% protection against mortality, but there was no protection when free PG-E was applied.

### 3.7. Effect of CI-Vesicles Containing PG-E in UV-Irradiated HaCaT Cells

**Figure 7:**
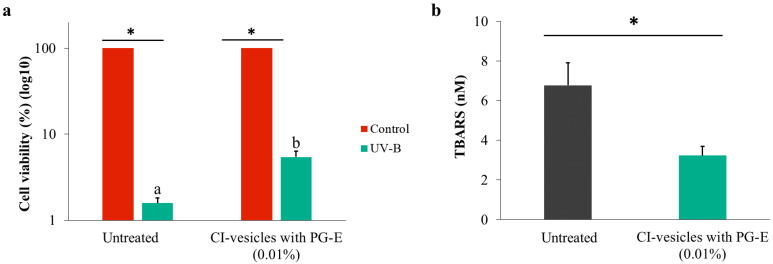
Viability of HaCaT cells after UV-B exposure, determined by the MTT assay. The results were normalized as a function of the control cytotoxicity (**a**). TBARS (thiobarbituric acid reactive substances, nM) was released into the culture medium by HaCaT cells (**b**). The HaCaT cells were exposed to UV radiation after treatment for 24 h with cauliflower inflorescence vesicles (CI-vesicles) with pomegranate extract (PG-E) (0.01%). Data are means ± SE (*n =* 6). Different letters indicate significant differences between groups. *, significant differences compared to the respective control (*p <* 0.05).

**Figure 8:**
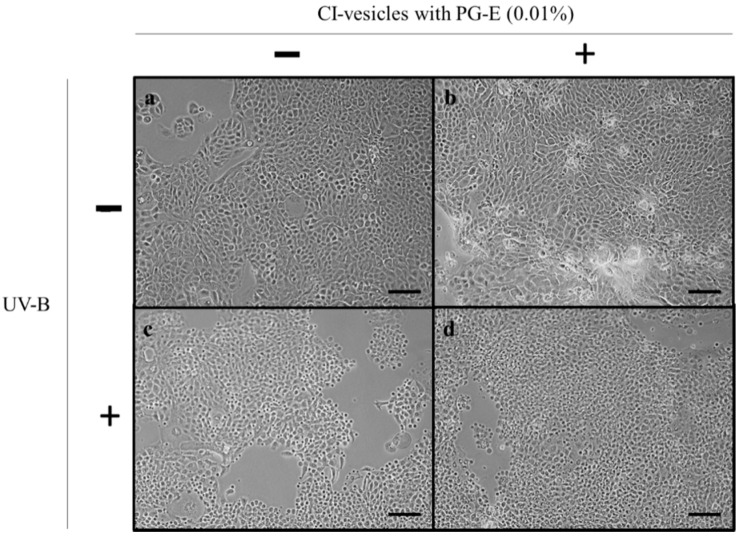
Phase-contrast microscopy images of HaCaT cells without cauliflower inflorescence vesicles (CI-vesicles) and UV-B treatment (**a**), with CI-vesicles and without UV-B treatment (**b**), without CI-vesicles and with UV-B treatment (**c**), and with CI-vesicles and UV-B treatment (**d**). Scale bars = 100 µm.

To investigate the effects of CI-vesicles containing PG-E on the proliferation of cells exposed to UV-B radiation, the MTT assay was performed with HaCaT cells. As shown in Figure 7a, the viability of UV-irradiated HaCaT cells was significantly decreased compared to non-irradiated cells. Cells treated with PG-E encapsulated in CI-vesicles prior to UV-B irradiation showed an increase in viability compared to untreated cells. Besides, the morphological changes induced by UV-B radiation are shown in Figure 8.

UV radiation causes oxidative stress in skin cells and the formation of free radicals and ROS, associated with lipid peroxidation in cell membranes. The ability of CI-vesicles with PG-E to decrease lipid peroxidation caused by UV radiation was assayed in HaCaT cells. The thiobarbituric acid reactive substances (TBARS) produced by HaCaT cells, as a by-product of lipid peroxidation, were analyzed inside of HaCaT cells (Figure S2) and in the culture medium (Figure 7b), since after UV radiation exposure, most of the TBARS are released by the cells [54]. For cells subjected to UV radiation, the 24-h PG-E pre-treatment significantly reduced the release of TBARS into the medium.

### 3.8. Effect of CI-Vesicles with PG-E on MtDNA Common Deletion in UV-Irradiated HaCaT Cells

**Figure 9:**
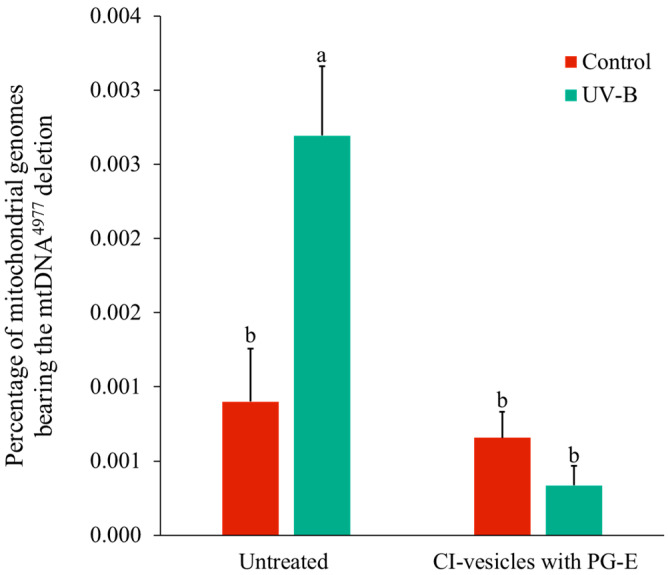
Amount of the “common deletion” 4977 in mitochondrial DNA (mtDNA^4977^) in HaCaT cells exposed to UV radiation without prior treatment or treated for 24 h with cauliflower inflorescence vesicles (CI-vesicles) with pomegranate extract (PG-E) (0.01%) before radiation exposure. Data are means ± SE (*n =* 3). Different letters indicate significant differences (*p <* 0.05).

The percentage of deleted mtDNA (mtDNA4977, “common deletion”) in HaCaT cells that were untreated or were treated with CI-vesicles containing PG-E (0.01%; 24 h) before being exposed to UV radiation was determined (Figure 9). Non-irradiated HaCaT cells displayed a small and similar amount of mtDNA4977 regardless of whether or not they had been treated with CI-vesicles containing PG-E. Cells that had not been treated with CI-vesicles containing PG-E showed an increase in the percentage of common deletion (mtDNA4977) when irradiated with UV, while cells treated with CI-vesicles containing PG-E for 24 h before UV radiation exposure showed an amount of mtDNA4977 comparable to that of non-irradiated control cells (Figure 9).

## 4. Discussion

Due to the variety of beneficial effects of pomegranate extract (PG-E) enriched in punicalagin [1,55], a vehicle is needed to improve its functionality in therapeutic and cosmetic applications, with the focus on its use as a protective agent against damaging environmental factors, such as UV radiation or pollution [56,57]. In the last few years, the use of vehicles or carriers from natural sources has been an object of study [58,59]; specifically, the use of membrane vesicles from plant materials as nanocarriers has shown promising results in different fields, such as cosmetics [28], medicine [26], or agriculture [35,36].

This work highlights the potential use of cauliflower inflorescence membrane vesicles (CI-vesicles) to encapsulate PG-E for cosmetic applications. The preparation of these vesicles consists of the purification of the plant membrane fraction, as described in Rios et al. [35], which allows a reproducible preparation of vesicles in terms of yield and size. The entrapment efficiency (EE) of the nanocarrier will depend on factors, such as the integrity and chemical composition of the vesicles, but also on the chemical and physical properties of the encapsulated compound [34,60]. In this work, an EE of 46.5% was obtained for PG-E encapsulated in CI-vesicles, similar to that reported previously with broccoli-derived plasma membrane vesicles, for which EE values around 50% were obtained when encapsulating two dyes [28]. Regarding other works where PG-E was encapsulated in nanocarriers, values of EE similar to that obtained in our study were reported. Marin et al. [61] showed an EE of 63% for a PG-E encapsulated in liposomes and established that punicalagin (a polar phenolic compound of PG-E) is located in the aqueous core of the liposomes, while ellagic acid intercalates into the aliphatic-chain zone of the membrane since it is poorly soluble in polar solvents. Besides, it is known that phenolic compounds interact with the polar head and also intercalate into the bilayer membrane [62]. This knowledge, together with the EE percentages, gives us an idea of how our system, based on membrane vesicles and a PG-E, is composed and arranged.

After obtaining acceptable EE data, the stability over time of the system (CI-vesicles with PG-E) was the next point to study. The stability was addressed on several fronts: protein content, color, and antioxidant activity. None of these parameters was affected by the passage of time (3 months) at any of the storage temperatures (20 °C, 4 °C, and 40 °C), which evidences the suitability of the system for its final purpose. The CI-vesicles with PG-E are maintained in a solution based on a polyalcohol protector, which promotes high stability during storage as it decreases the surface tension of water [63]. In this way, the polyalcohol also protects the non-encapsulated extract. This type of assay is an accelerated storage-stability test and is commonly used to determine the long-term behavior of the system [64]. Other studies also demonstrated that the antioxidant activity of materials derived from pomegranate persists over time without changes. Mali et al. [65] showed that the antioxidant activity of pomegranate peel powder had not changed after 90 days at room temperature, but degradation had occurred in aqueous solutions. The color stability is related to oxidation, with the concomitant implications for cell functionality. Therefore, our stability assays showed that this is a system suitable for use in further cosmeceutical applications.

The protective effect of PG-E encapsulated in CI-vesicles was assayed in a keratinocyte cell line (HaCaT). Keratinocytes form the majority of the epidermis, the outermost layer of the skin; therefore, these cells are part of the first defense barrier against harmful external stimuli, such as UV radiation or pollution [66]. The cytotoxicity of CI-vesicles containing PG-E was assayed at different concentrations to find the working concentration. Application of the highest concentration induced a significant decrease in viability with respect to the control, but in such trials, cell viabilities around 80% are not considered to represent cytotoxicity [53]. Hence, this concentration was chosen to ensure clear effects in subsequent assays. This cytotoxicity was due to the CI-vesicles because free PG-E did not show cytotoxicity and even increased cell viability. There are no previous reports of cytotoxicity caused by this type of membrane vesicle, and several works have reported that such vesicles have zero cytotoxicity at suitable concentrations in cell cultures [67,68]. In other studies, null cytotoxicity of PG-E has been reported for HaCaT cells; for example, in Liu et al. [13], for concentrations of PG-E from 6.25 to 100 µg/mL. In our work, the highest concentration of PG-E was 13 µg/mL; thus, our results are in line with what has been reported previously. These good results obtained in the cytotoxicity tests, together with previous results from our group, confirm the suitability of the system. Previous work [28] showed an interaction between plant and human cell membranes, with plasma membrane vesicles from broccoli exhibiting a high fusion ability with human keratinocytes.

The protective effect of our system (PG-E encapsulated in CI-vesicles) against heavy metals and UV-B radiation was determined in this work. Heavy metals like lead and mercury are common air pollutants and have been shown to trigger health risks [69]. To analyze the effectiveness of CI-vesicles containing PG-E with regard to decreasing the damage caused by heavy metals in keratinocytes, different concentrations of lead acetate and mercuric chloride and a mixture of both were applied to HaCaT cells. Individually, both metals caused a reduction in cell viability (data not shown), as reported previously in other studies [70,71], but in this work, assays were also carried out with a mixture of lead acetate and mercuric chloride. An important reduction in cell viability was shown after 24 h of incubation of HaCaT cells with different concentrations of the metals and in the same way as previously reported, the reduction being highly dose-dependent [71]. Oxidative stress has been revealed as one of the actors in heavy metal-induced cytotoxicity [18,72,73]; thus, due to their antioxidant capacity, the protective effects of CI-vesicles with PG-E against heavy metals were tested. As described in the Results section, an improvement in cell viability appeared when PG-E was applied in encapsulated form. This could be due to a better entry of the extract into the cells when it is encapsulated due to the fusion of the vesicles with the cell membranes [28]. Studies were carried out to determine the effectiveness of CI-vesicles with PG-E against UV-B radiation (290–320 nm), which is the main UV component that causes a wide variety of skin disorders, including skin cancers [74]. The cell viability after irradiation with UV-B was higher when CI-vesicles with PG-E were applied 24 h before irradiation. In addition, the TBARS release into the medium after irradiation with UV-B was lower for cells previously treated with PG-E encapsulated in CI-vesicles than for untreated cells, and this response is probably related to the antioxidant capacity of the PG-E. A cellular environment with increased lipid peroxidation can produce immune and inflammatory responses [75]. The reduction of these inflammatory responses, thereby maintaining or restoring cell homeostasis, could be achieved with our PG-E treatment. The results obtained in our experiments showed that the encapsulated PG-E provided protective effects against the UV-B-induced oxidative stress, suggesting prevention of membrane damage. In this way, our results with encapsulated PG-E are similar to those obtained in other work with free pomegranate extracts but using higher concentrations [76]. These previous studies also showed promising results regarding protection against UV-B radiation: the protection by CI-vesicles with PG-E against the oxidative stress triggered by UV-B radiation in HaCaT cells coincided with higher cell viability, an enhanced intracellular glutathione (GSH) content, and a decline in membrane damage (analyzed as lipid peroxidation). Besides, in other work, inhibition of UV-B-mediated activation and phosphorylation of the mitogen-activated protein kinase (MAPK) and factor nuclear kappa B (NF-κB) pathways by a pomegranate fruit extract was revealed [77].

Our results showed a protective effect of CI-vesicles with PG-E against the mutations in mtDNA induced by UV-B radiation, measured as common deletion mtDNA^4997^. These types of mutations are considered as markers for ROS-mediated genotoxicity [78], and several previous studies have shown an increase in these mutations (deletions) in the mtDNA of cells exposed to UV-B radiation [48,50]. This is in accordance with our results because an increase in mtDNA^4997^ in HaCaT cells that were not treated with the protective agent was found after UV irradiation. The antioxidant capacity of PG-Es could play an important role in preventing the deletion since, as we stated above, this mutation is related to an increase in ROS generation. In this sense, melatonin, an endogenous antioxidant, has been reported to prevent mtDNA^4997^ under both basal conditions and induced oxidative stress in cybrids [79]. Our results, along with others published previously [77,80], support the capacity of antioxidant compounds to prevent damage related to aging and UV-B exposure [81]. The fact that the protective effect was higher for encapsulated PG-E could be due to its greater penetrability in cells, as reported previously [28].

## 5. Conclusions

We have shown that vesicles derived from cauliflower inflorescences could serve as nanocarriers for PG-E by demonstrating their stability and their relatively high entrapment efficiency. The results for keratinocyte culture cells (HaCaT) show the non-cytotoxicity of the system (PG-E encapsulated in CI-vesicles) and its protection against heavy metals and UV-B radiation through a reduction of oxidative stress—which entails a decrease in membrane damage due to a reduction of lipid peroxidation and the prevention of mutations in DNA, such as deletions in mitochondrial DNA. The HaCaTs, a spontaneously immortalized human keratinocyte cell line, have been used widely to investigate different toxicities and molecular mechanisms related to skin, and, therefore, the results obtained with this cell line can reliably be extrapolated to what could happen in real skin. Thereby, this work represents a further advance in the use of vesicles obtained from plant material as nanocarriers for topical applications, with promising results for the encapsulation of antioxidant extracts; in this case, PG-E, which protected keratinocyte cells from UV and heavy metal damage at very low concentrations.

## Figures and Tables

**Table 1:** Primer sets used for quantitative PCR; a from Li [49], b and c from Koch [48].

Target Name	Product Size	Forward Primer (5′ → 3′)	Reverse Primer (5′ → 3′)
Beta-actin ^a^	205 bp	GGCGGCAACACCATGTACCCT	AGGGGCCGGACTCGTCATACT
Total mtDNA ^b^	83 bp	GATTTGGGTACCACCCAAGTATT	AATATTCATGGTGGCTGGCAGTA
Common deletion mtDNA^4977 c^	107 bp	ACCCCCATACTCCTTACACTATTCC	AAGGTATTCCTGCTAATGCTAGGCT

**Table 2:** Characteristics of CI-vesicles, CI-vesicles with PG-E, and PG-E: Particle Size, Polydispersity Index, and Zeta Potential.

	CI-Vesicles	CI-Vesicles with PG-E	PG-E
Z-average (nm)	620.72 ± 25.17 a	797.50 ± 38.93 b	-
Polydispersity index (0–1)	0.70 ± 0.03 a	0.76 ± 0.12 a	-
Z-potential (mV)	−21.56 ± 0.38 a	−21.65 ± 0.24 a	−15.04 ± 0.40 b

PG-E: pomegranate extract, CI-vesicles: cauliflower inflorescence vesicles. Data are means ± SE (*n =* 3). Different letters indicate significant differences between groups for each variable.

**Table 3:** Entrapment efficiency calculated from absorbance data and the protein content (mg) in samples before and after passage through a Sephadex column and fractions collection.

	Free PG-E	CI-Vesicles with PG-E
The total area under the curve (a.u.)	3160 ± 33.20	3357 ± 161.40
Encapsulated area (a.u.)	-	1561 ± 234.15
Entrapment efficiency (%)	-	46.50 ± 1.62
Protein before column (mg)	0	0.22 ± 0.02
Total protein collected (mg)	0	0.21 ± 0.01

PG-E: pomegranate extract, CI-vesicles: cauliflower inflorescence vesicles, a.u.: arbitrary unit. Data are means ± SE (*n* = 3).

**Table 4:** Antioxidant activity (µM TE) of cauliflower inflorescence vesicles (CI-vesicles) and pomegranate extract (PG-E), free or encapsulated in CI-vesicles.

	Antioxidant Activity (µM TE)
CI-vesicles	0
PG-E, free	5830.25 ± 169.00
CI-vesicles with PG-E	5786.29 ± 148.00

TE: Trolox Equivalents. Data are means ± SE (*n =* 3).

**Table 5:** The protection provided by free pomegranate extract (PG-E) and PG-E encapsulated in cauliflower vesicles (CI-vesicles) against heavy metals (1000 µM lead acetate + 70 µM mercuric chloride) in HaCaT cells.

	CI-Vesicles with PG-E		Free PG-E	
Cell Viability (%)	Untreated	CI-vesicles with PG-E	Untreated	Free PG-E
	25.74 ± 1.71	43.22 ± 3.97	25.74 ± 1.71	23.89 ± 2.47
Viability improvement (%)	74.40 ± 22.14		−5.11 ± 11.94	
Mortality protection (%)	27.13 ± 6.31		−2.80 ± 4.24	

Data are means ± SE (*n =* 6).

## Data Availability

The data presented in this study are available on request from the corresponding author.

## Supplementary Materials

The following are available online at https://www.mdpi.com/1999-4923/13/2/271/s1, Figure S1: TEM images of CI-vesicles. Figure S2: TBARS (nM/mg protein) determined in HaCaT cells. Table S1: Antioxidant activity (µM TE) of pomegranate extract (PG-E), free or encapsulated in CI-vesicles, after released with methanol and chloroform.

## References

[1] Jurenka J. (2008). Therapeutic Applications of Pomegranate (*Punica granatum* L.): A Review. Altern. Med. Rev..

[2] Gil M.I., Tomas-Barberan F.A., Hess-Pierce B., Holcroft D.M., Kader A.A. (2000). Antioxidant Activity of Pomegranate Juice and Its Relationship with Phenolic Composition and Processing. J. Agric. Food Chem..

[3] Russo M., Fanali C., Tripodo G., Dugo P., Muleo R., Dugo L., de Gara L., Mondello L. (2018). Analysis of Phenolic Compounds in Different Parts of Pomegranate (Punica granatum) Fruit by HPLC-PDA-ESI/MS and Evaluation of Their Antioxidant Activity: Application to Different Italian Varieties. Anal. Bioanal. Chem..

[4] Liu G., Xu X., Hao Q., Gao Y. (2009). Supercritical CO2 Extraction Optimization of Pomegranate (*Punica granatum* L.) Seed Oil Using Response Surface Methodology. LWT Food Sci. Technol..

[5] Hernández F., Melgarejo P., Tomás-Barberán F.A., Artés F. (1999). Evolution of Juice Anthocyanins during Ripening of New Selected Pomegranate (*Punica granatum*) Clones. Eur. Food Res. Technol..

[6] Espín J.C., García-Conesa M.T., Tomás-Barberán F.A. (2007). Nutraceuticals: Facts and Fiction. Phytochemistry.

[7] Katz S.R., Newman R.A., Lansky E.P. (2007). Punica granatum: Heuristic Treatment for Diabetes Mellitus. J. Med. Food.

[8] Lansky E.P., Newman R.A. (2007). Punica granatum (pomegranate) and Its Potential for Prevention and Treatment of Inflammation and Cancer. J. Ethnopharmacol..

[9] Sohrab G., Nasrollahzadeh J., Zand H., Amiri Z., Tohidi M., Kimiagar M. (2014). Effects of Pomegranate Juice Consumption on Inflammatory Markers in Patients with Type 2 Diabetes: A Randomized, Placebo-Controlled Trial. J. Res. Med. Sci..

[10] Hosseini B., Saedisomeolia A., Wood L.G., Yaseri M., Tavasoli S. (2016). Effects of Pomegranate Extract Supplementation on Inflammation in Overweight and Obese Individuals: A Randomized Controlled Clinical Trial. Complementary Ther. Clin. Pract..

[11] Hossin F.L.A. (2009). Effect of Pomegranate (Punica granatum) Peels and It’s Extract on Obese Hypercholesterolemic Rats. Pak. J. Nutr..

[12] Afaq F., Zaid M.A., Khan N., Dreher M., Mukhtar H. (2009). Protective Effect of Pomegranate-Derived Products on UVB-Mediated Damage in Human Reconstituted skin. Exp. Dermatol..

[13] Liu C., Guo H., DaSilva N.A., Li D., Zhang K., Wan Y., Gao X.-H., Chen H.-D., Seeram N.P., Ma H. (2019). Pomegranate (Punica granatum) Phenolics Ameliorate Hydrogen Peroxide-Induced Oxidative Stress and Cytotoxicity in Human Keratinocytes. J. Funct. Foods.

[14] Baroni A., Buommino E., de Gregorio V., Ruocco E., Ruocco V., Wolf R. (2012). Structure and Function of the Epidermis Related to Barrier Properties. Clin. Dermatol..

[15] D’Orazio J., Jarrett S., Amaro-Ortiz A., Scott T. (2013). UV Radiation and the Skin. Int. J. Mol. Sci..

[16] Kappus H., Reinhold C. (1994). Heavy Metal-Induced Cytotoxicity to Cultured Human Epidermal Keratinocytes and Effects of Antioxidants. Toxicol. Lett..

[17] Aitken G.R., Henderson J.R., Chang S.C., McNeil C.J., Birch-Machin M.A. (2007). Direct Monitoring of UV-Induced Free Radical Generation in HaCaT Keratinocytes. Clin. Exp. Dermatol..

[18] Nzengue Y., Steiman R., Garrel C., Lefèbvre E., Guiraud P. (2008). Oxidative Stress and DNA Damage Induced by Cadmium in the Human Keratinocyte HaCaT Cell Line: Role of Glutathione in the Resistance to Cadmium. Toxicology.

[19] Cooke M.S., Evans M.D., Dizdaroglu M., Lunec J. (2003). Oxidative DNA Damage: Mechanisms, Mutation, and Disease. FASEB J..

[20] Yakes F.M., Chen Y., van Houten B., Pfeifer G.P. (1996). PCR-Based Assays for the Detection and Quantitation of DNA Damage and Repair. BT-Technologies for Detection of DNA Damage and Mutations.

[21] Shoffner J.M., Lott M.T., Voljavec A.S., Soueidan S.A., Costigan D.A., Wallace D.C. (1989). Spontaneous Kearns-Sayre/Chronic External Ophthalmoplegia Plus Syndrome Associated with a Mitochondrial DNA Deletion: A Slip-Replication Model and Metabolic Therapy. Proc. Natl. Acad. Sci. USA.

[22] Jaeger A., Weiss D.G., Jonas L., Kriehuber R. (2012). Oxidative Stress-Induced Cytotoxic and Genotoxic Effects of Nano-Sized Titanium Dioxide Particles in Human HaCaT Keratinocytes. Toxicology.

[23] Pacheco-Palencia L.A., Noratto G., Hingorani L., Talcott S.T., Mertens-Talcott S.U. (2008). Protective Effects of Standardized Pomegranate (*Punica granatum* L.) Polyphenolic Extract in Ultraviolet-Irradiated Human Skin Fibroblasts. J. Agric. Food Chem..

[24] Ventura J., Alarcón-Aguilar F., Roman-Ramos R., Campos-Sepulveda E., Reyes-Vega M.L., Daniel Boone-Villa V., Jasso-Villagómez E.I., Aguilar C.N. (2013). Quality and Antioxidant Properties of a Reduced-Sugar Pomegranate Juice Jelly with an Aqueous Extract of Pomegranate Peels. Food Chem..

[25] Çam M., Içyer N.C., Erdoǧan F. (2014). Pomegranate Peel Phenolics: Microencapsulation, Storage Stability and Potential Ingredient for Functional Food Development. LWT Food Sci. Technol..

[26] Ju S., Mu J., Dokland T., Zhuang X., Wang Q., Jiang H., Xiang X., Deng Z.B., Wang B., Zhang L. (2013). Grape Exosome-Like Nanoparticles Induce Intestinal Stem Cells and Protect Mice from DSS-Induced Colitis. Mol. Ther..

[27] György B., Hung M.E., Breakefield X.O., Leonard J.N. (2015). Therapeutic Applications of Extracellular Vesicles: Clinical Promise and Open Questions. Annu. Rev. Pharmacol. Toxicol..

[28] Yepes-Molina L., Martínez-Ballesta M.C., Carvajal M. (2020). Plant Plasma Membrane Vesicles Interaction with Keratinocytes Reveals Their Potential as Carriers. J. Adv. Res..

[29] Peng L.-H., Wang M.-Z., Chu Y., Zhang L., Niu J., Shao H.-T., Yuan T.-J., Jiang Z.-H., Gao J.-Q., Ning X.-H. (2020). Engineering Bacterial Outer Membrane Vesicles as Transdermal Nanoplatforms for Photo-TRAIL—Programmed Therapy Against Melanoma. Sci. Adv..

[30] Zhang M., Viennois E., Xu C., Merlin D. (2016). Plant Derived Edible Nanoparticles as a New Therapeutic Approach Against Diseases. Tissue Barriers.

[31] Martínez Ballesta M.C., Pérez-Sánchez H., Moreno D.A., Carvajal M. (2016). Plant Plasma Membrane Aquaporins in Natural Vesicles as Potential Stabilizers and Carriers of Glucosinolates. Colloids Surf. B Biointerfaces.

[32] Martínez Ballesta M.C., García-Gomez P., Yepes-Molina L., Guarnizo A.L., Teruel J.A., Carvajal M. (2018). Plasma Membrane Aquaporins Mediates Vesicle Stability in Broccoli. PLoS ONE.

[33] Garcia-Ibañez P., Nicolas-Espinosa J., Carvajal M. (2021). Plasma Membrane Vesicles from Cauliflower Meristematic Tissue and Their Role in Water Passage. BMC Plant Biol..

[34] Danaei M., Dehghankhold M., Ataei S., Hasanzadeh Davarani F., Javanmard R., Dokhani A., Khorasani S., Mozafari M.R. (2018). Impact of Particle Size and Polydispersity Index on the Clinical Applications of Lipidic Nanocarrier Systems. Pharmaceutics.

[35] Rios J.J., Garcia-Ibañez P., Carvajal M. (2019). The Use of Biovesicles to Improve the Efficiency of Zn Foliar Fertilization. Colloids Surf. B Biointerfaces.

[36] Rios J.J., Yepes-Molina L., Martinez-Alonso A., Carvajal M. (2020). Nanobiofertilization as a Novel Technology for Highly Efficient Foliar Application of Fe and B in Almond Trees. R. Soc. Open Sci..

[37] Bradford M.M. (1976). A Rapid and Sensitive Method for the Quantitation of Microgram Quantities of Protein Utilizing the Principle of Protein-Dye Binding. Anal. Biochem..

[38] Karsch-Bluman A., Avraham S., Assayag M., Schwob O., Benny O. (2019). Encapsulated Carbenoxolone Reduces Lung Metastases. Cancers.

[39] Hirai Y., Terashima T., Takenaka M., Sawamoto M. (2016). Precision Self-Assembly of Amphiphilic Random Copolymers into Uniform and Self-Sorting Nanocompartments in Water. Macromolecules.

[40] Ben Yehuda Greenwald M., Ben Sasson S., Bianco-Peled H. (2013). A New Method for Encapsulating Hydrophobic Compounds within Cationic Polymeric Nanoparticles. J. Microencapsul..

[41] Yepes-Molina L., Carvajal M., Martínez-Ballesta M.C. (2020). Detergent Resistant Membrane Domains in Broccoli Plasma Membrane Associated to the Response to Salinity Stress. Int. J. Mol. Sci..

[42] Schindelin J., Arganda-Carreras I., Frise E., Kaynig V., Longair M., Pietzsch T., Preibisch S., Rueden C., Saalfeld S., Schmid B. (2012). Fiji: An Open-Source Platform for Biological-Image Analysis. Nat. Methods.

[43] Blois M.S. (1958). Antioxidant Determinations by the Use of a Stable Free Radical. Nature.

[44] Bondet V., Brand-Williams W., Berset C. (1997). Kinetics and Mechanisms of Antioxidant Activity Using the DPPH**·** Free Radical Method. LWT Food Sci. Technol..

[45] Boukamp P., Petrussevska R.T., Breitkreutz D., Hornung J., Markham A., Fusenig N.E. (1988). Normal Keratinization in a Spontaneously Immortalized Aneuploid Human Keratinocyte Cell Line. J. Cell Biol..

[46] Mosmann T. (1983). Rapid Colorimetric Assay for Cellular Growth and Survival: Application to Proliferation and Cytotoxicity Assays. J. Immunol. Methods.

[47] Fraga C.G., Leibovitz B.E., Tappel A.L. (1988). Lipid Peroxidation Measured as Thiobarbituric Acid-Reactive Substances in Tissue Slices: Characterization and Comparison with Homogenates and Microsomes. Free Radic. Biol. Med..

[48] Koch H., Wittern K.P., Bergemann J. (2001). In Human Keratinocytes the Common Deletion Reflects Donor Variabilities Rather Than Chronologic Aging and Can Be Induced by Ultraviolet a Irradiation. J. Invest. Dermatol..

[49] Li L., Yan Y., Xu H., Qu T., Wang B. (2011). Selection of Reference Genes for Gene Expression Studies in Ultraviolet B-Irradiated Human Skin Fibroblasts Using Quantitative Real-Time PCR. BMC Mol. Biol..

[50] Powers J.M., Murphy G., Ralph N., O’Gorman S.M., Murphy J.E.J. (2016). Mitochondrial DNA Deletion Percentage in Sun Exposed and Non Sun Exposed Skin. J. Photochem. Photobiol. B Biol..

[51] R Core Team (2018). R: A Language and Environment for Statistical Computing.

[52] Vallar S., Houivet D., El Fallah J., Kervadec D., Haussonne J.M. (1999). Oxide Slurries Stability and Powders Dispersion: Optimization with Zeta Potential and Rheological Measurements. J. Eur. Ceram. Soc..

[53] Standard I. (2009). Biological Evaluation of Medical Devices—Part 5: Tests for in Vitro Cytotoxicity. Geneve Switz. Int. Organ. Stand..

[54] D’Angelo S., Ingrosso D., Migliardi V., Sorrentino A., Donnarumma G., Baroni A., Masella L., Tufano M.A., Zappia M., Galletti P. (2005). Hydroxytyrosol, a Natural Antioxidant from Olive Oil, Prevents PROTEin Damage Induced by Long-Wave Ultraviolet Radiation in Melanoma Cells. Free Radic. Biol. Med..

[55] Sorrenti V., Randazzo C.L., Caggia C., Ballistreri G., Romeo F.V., Fabroni S., Timpanaro N., Raffaele M., Vanella L. (2019). Beneficial Effects of Pomegranate Peel Extract and Probiotics on Pre-Adipocyte Differentiation. Front. Microbiol..

[56] Marabini L., Melzi G., Lolli F., Dell’Agli M., Piazza S., Sangiovanni E., Marinovich M. (2020). Effects of *Vitis Vinifera* L. Leaves Extract on UV Radiation Damage in Human Keratinocytes (HaCaT). J. Photochem. Photobiol. B Biol..

[57] Rajnochová Svobodová A., Gabrielová E., Ulrichová J., Zálešák B., Biedermann D., Vostálová J. (2019). A Pilot Study of the UVA-Photoprotective Potential of Dehydrosilybin, Isosilybin, Silychristin, and Silydianin on Human Dermal Fibroblasts. Arch. Dermatol. Res..

[58] Lohani A., Verma A. (2017). Vesicles: Potential Nano Carriers for the Delivery of Skin Cosmetics. J. Cosmet. Laser Ther..

[59] Voronin D., Vikulina A., Voronin D., Fakhrullin R., Vinokurov V., Volodkin D. (2020). Naturally Derived Nano- and Micro-Drug Delivery Vehicles: Halloysite, Vaterite and Nanocellulose. New J. Chem..

[60] Tan C., Xue J., Lou X., Abbas S., Guan Y., Feng B., Zhang X., Xia S. (2014). Liposomes as Delivery Systems for Carotenoids: Comparative Studies of Loading Ability, Storage Stability and in Vitro Release. Food Funct..

[61] Marín D., Alemán A., Sánchez-Faure A., Montero P., Gómez-Guillén M.C. (2018). Freeze-Dried Phosphatidylcholine Liposomes Encapsulating Various Antioxidant Extracts from Natural Waste as Functional Ingredients in Surimi Gels. Food Chem..

[62] Pawlikowska-Pawlȩga B., Dziubińska H., Król E., Trȩbacz K., Jarosz-Wilkołazka A., Paduch R., Gawron A., Gruszecki W.I. (2014). Characteristics of Quercetin Interactions with Liposomal and Vacuolar Membranes. Biochim. Biophys. Acta Biomembr..

[63] Gekko K., Timasheff S.N. (1981). Mechanism of Protein Stabilization by Glycerol: Preferential Hydration in Glycerol-Water Mixtures. Biochemistry.

[64] Robert P., Gorena T., Romero N., Sepulveda E., Chavez J., Saenz C. (2010). Encapsulation of Polyphenols and Anthocyanins from Pomegranate (Punica granatum) by Spray Drying. Int. J. Food Sci. Technol..

[65] Mali A.B., Khedkar K., Lele S.S. (2011). Effect of Gamma Irradiation on Total Phenolic Content and in Vitro Antioxidant Activity of Pomegranate (*Punica Granatum* L.) Peels. Food Nutr. Sci..

[66] Schäfer M., Werner S. (2011). The Cornified Envelope: A First Line of Defense against Reactive Oxygen Species. J. Invest. Dermatol..

[67] Sinha A., Suresh P.K. (2019). Enhanced Induction of Apoptosis in HaCaT Cells by Luteolin Encapsulated in PEGylated Liposomes—Role of Caspase-3/Caspase-14. Appl. Biochem. Biotechnol..

[68] Díaz C., Vargas E., Gätjens-Boniche O. (2006). Cytotoxic effect induced by retinoic acid loaded into galactosyl-sphingosine containing liposomes on human hepatoma cell lines. Int. J. Pharm..

[69] World Health Organization (2007). Health Risks of Heavy Metals from Long-Range Transboundary Air Pollution.

[70] Hwang T.-L., Chen H.-Y., Changchien T.-T., Wang C.-C., Wu C.-M. (2013). The Cytotoxicity of Mercury Chloride to the Keratinocytes is Associated with Metallothionein Expression. Biomed. Rep..

[71] Bae D.S., Gennings C., Carter W.H., Yang R.S.H., Campain J.A. (2001). Toxicological Interactions among Arsenic, Cadmium, Chromium, and Lead in Human Keratinocytes. Toxicol. Sci..

[72] Ahmad S., Mahmood R. (2019). Mercury Chloride Toxicity in Human Erythrocytes: Enhanced Generation of ROS and RNS, Hemoglobin Oxidation, Impaired Antioxidant Power, and Inhibition of Plasma Membrane Redox System. Environ. Sci. Pollut. Res..

[73] Patra R.C., Rautray A.K., Swarup D. (2011). Oxidative Stress in Lead and Cadmium Toxicity and Its Amelioration. Vet. Med. Int..

[74] Anna B., Blazej Z., Jacqueline G., Andrew C.J., Jeffrey R., Andrzej S. (2007). Mechanism of UV-Related Carcinogenesis and Its Contribution to Nevi/Melanoma. Expert Rev. Dermatol..

[75] Halliday G.M. (2005). Inflammation, Gene Mutation and Photoimmunosuppression in Response to UVR-Induced Oxidative Damage Contributes to Photocarcinogenesis. Mutat. Res. Fundam. Mol. Mech. Mutagen..

[76] Zaid M.A., Afaq F., Syed D.N., Dreher M., Mukhtar H. (2007). Inhibition of UVB-Mediated Oxidative Stress and Markers of Photoaging in Immortalized HaCaT Keratinocytes by Pomegranate Polyphenol Extract POMx. Photochem. Photobiol..

[77] Afaq F., Malik A., Syed D., Maes D., Matsui M.S., Mukhtar H. (2005). Pomegranate Fruit Extract Modulates UVB-Mediated Phosphorylation of Mitogen Activated Protein Kinases and Activation of Nuclear Factor Kappa B in Normal Human Epidermal Keratinocytes. Photochem. Photobiol..

[78] Birch-Machin M.A., Tindall M., Turner R., Haldane F., Rees J.L. (1998). Mitochondrial DNA Deletions in Human Skin Reflect Photo- Rather Than Chronologic Aging. J. Invest. Dermatol..

[79] Jou M.J., Peng T.I., Yu P.Z., Jou S.B., Reiter R.J., Chen J.Y., Wu H.Y., Chen C.C., Hsu L.F. (2007). Melatonin Protects Against Common Deletion of Mitochondrial DNA-Augmented Mitochondrial Oxidative Stress and Apoptosis. J. Pineal Res..

[80] Tavanai E., Mohammadkhani G. (2017). Role of Antioxidants in Prevention of Age-Related Hearing Loss: A Review of Literature. Eur. Arch. Oto Rhino Laryngol..

[81] Dunaway S., Odin R., Zhou L., Ji L., Zhang Y., Kadekaro A.L. (2018). Natural Antioxidants: Multiple Mechanisms to Protect Skin from Solar Radiation. Front. Pharmacol..

