# Supplementary material for: Nanoencapsulation of Pomegranate Extract to Increase Stability and Potential Dermatological Protection
Source: Pharmaceutics. 2021 Feb 17;13(2):271. doi: 10.3390/pharmaceutics13020271 (PMC7922654; doi:10.3390/pharmaceutics13020271)
Supplement: Supplementary file 1 [file pharmaceutics-13-00271-s001.pdf]

pharmaceutics

Supplementary Materials: Nanoencapsulation of Pomegranate Extract to Increase Stability and Potential Dermatological Protection

Lucía Yepes-Molina, Jose A. Hernández and Micaela Carvajal

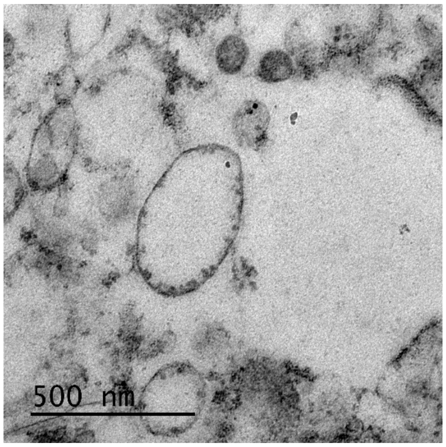

Figure S1. Transmission electronic microscopy image of CI-vesicles.

Table S1. Antioxidant activity (μM TE) of pomegranate extract (PG-E), free or encapsulated in CI-vesicles after released with methanol and chloroform.

	**Antioxidant activity (μM TE)**
PG-E, free	5830.25 ± 169.00
CI-vesicles with PG-E released with methanol	5786.29 ± 148.00
CI-vesicles with PG-E released with chloroform	5645.38 ± 331.45

TE: Trolox Equivalents. Data are means ± SE (n = 3).

Copyright: © 2021 by the authors. Licensee MDPI, Basel, Switzerland. This article is an open access article distributed under the terms and conditions of the Creative Commons Attribution (CC BY) license (http://creativecommons.org/licenses/by/4.0/).

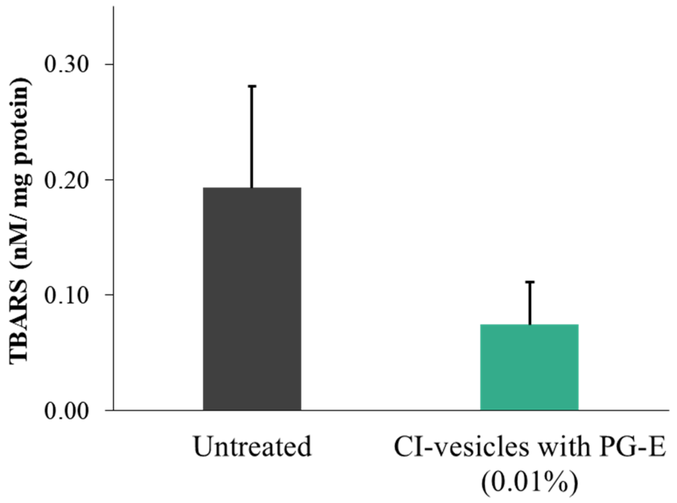

Figure S2. TBARS (nM/mg protein) determined in HaCaT cells exposed to UV radiation after treatment for 24 h with cauliflower inflorescence vesicles (CI-vesicles) with pomegranate extract (PG-E) (0.01%). Data are means ± SE (n = 6).
